# Supplementary material for: Influence of oral administration of kukoamine A on blood pressure in a rat hypertension model
Source: PLoS One. 2022 May 6;17(5):e0267567. doi: 10.1371/journal.pone.0267567 (PMC9075663; doi:10.1371/journal.pone.0267567)
Supplement: Attachment — Submitted filename: Comments of Manuscript ID PONE-D-21-35268.pdf [file pone.0267567.s002.pdf]

Manuscript ID: PONE-D-21-35268

This topic **"Influence of oral administration of kukoamine A on blood pressure in a rat hypertension model"** is an interesting work and quite good information.

However, before accepting publication, **minor revision is required** and some points in this manuscript need to clarify.

Minor comments are below…

1. For 14-day acute oral safety study section: W why the researcher uses female mice to study this effect? Generally, the animal's sexuality has influenced the genetic transformation or variance of a hormone than a male animal, so it will be difficult to control that factor and may influence to blood pressure determination?
   However, researcher use the male or female mice in this study? because in Line 145 mentioned difference mice gender?

2. Also, the time of oral test is quite short time?? Only 2 weeks of an animal experiment may not be enough to get the cover results about the blood parameter index changing.?

3. In discussion point Line 250, previous studies already showed that higher dose oral test in mice 25 and 50 mg kg-1 was safe, so why researcher try to study the only a lower concentration of kukoamine A?? in which 5 and 10 mg kg-1 seems to be safe for sure

4. Check all the error typing and make a correction

5. Suggestion: The present study was determined only physiological and blood characteristics of the animal model, however, to confirm the mechanisms of effects of kukoamine A on vascular function, other biomarkers need to determine such as the expression of the gene-related vascular system and the significant protein expression that play a role to control the blood pressure or hypertension pathway in the mammalian.
